# Arsenate removal from drinking water using by-products from conventional iron oxyhydroxides production as adsorbents coupled with submerged microfiltration unit

**DOI:** 10.1007/s11356-020-08327-w

**Published:** 2020-04-10

**Authors:** Muhammad Usman, Ioannis Katsoyiannis, Josma Henna Rodrigues, Mathias Ernst

**Affiliations:** 1 Institute for Water Resources and Water Supply, Hamburg University of Technology, Am Schwarzenberg-Campus 3, 20173 Hamburg, Germany; 2 Department of Chemistry, Laboratory of Chemical and Environmental Technology, Aristotle University of Thessaloniki, 54124 Thessaloniki, Greece

**Keywords:** Arsenic removal, Granular ferric hydroxide, Micro-sized iron oxyhydroxides, Waste utilization, Adsorption kinetics, Submerged membrane adsorption hybrid system, Drinking water production

## Abstract

Arsenic is among the major drinking water contaminants affecting populations in many countries because it causes serious health problems on long-term exposure. Two low-cost micro-sized iron oxyhydroxide-based adsorbents (which are by-products of the industrial production process of granular adsorbents), namely, micro granular ferric hydroxide (μGFH) and micro tetravalent manganese feroxyhyte (μTMF), were applied in batch adsorption kinetic tests and submerged microfiltration membrane adsorption hybrid system (SMAHS) to remove pentavalent arsenic (As(V)) from modeled drinking water. The adsorbents media were characterized in terms of iron content, BET surface area, pore volume, and particle size. The results of adsorption kinetics show that initial adsorption rate of As(V) by μTMF is faster than μGFH. The SMAHS results revealed that hydraulic residence time of As(V) in the slurry reactor plays a critical role. At longer residence time, the achieved adsorption capacities at As(V) permeate concentration of 10 μg/L (WHO guideline value) are 0.95 and 1.04 μg/mg for μGFH and μTMF, respectively. At shorter residence time of ~ 3 h, μTMF was able to treat 1.4 times more volumes of arsenic-polluted water than μGFH under the optimized experimental conditions due to its fast kinetic behavior. The outcomes of this study confirm that micro-sized iron oyxhydroxides, by-products of conventional adsorbent production processes, can successfully be employed in the proposed hybrid water treatment system to achieve drinking water guideline value for arsenic, without considerable fouling of the porous membrane.

**Figure:**
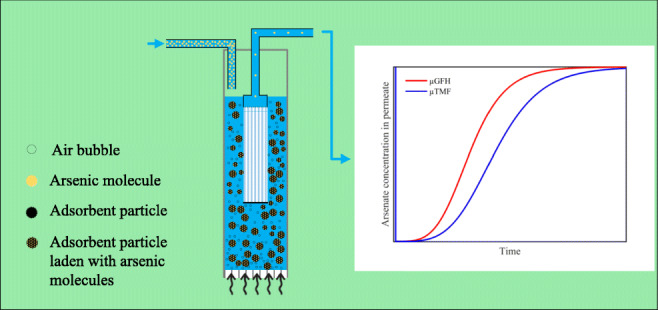
Graphical abstract

Graphical abstract

## Introduction

Groundwater is globally the foremost source of drinking water for human consumption. Arsenic contamination of drinking waters across the world is one of the most serious water related problems, because it affects big parts of the global population and it is very harmful to human health. It is well-known that inorganic forms of arsenic are a strong human carcinogen. The World Health Organization (WHO) has set a guideline value of 10 μg/L in drinking water. Both natural and anthropogenic sources contribute to elevated concentrations of arsenic in natural environments (Violante et al. 2006). In groundwaters, arsenic is present mainly with its inorganic forms, arsenite, which is the trivalent form of arsenic [As (III)], and arsenate, which is the pentavalent form of arsenic [As(V)]. More than 150 million inhabitants are under high health risk in more than 70 countries due to pollution of drinking water by arsenic (Abejón and Garea 2015). Therefore, many different attempts such as the use of zero valent iron with main applications in South east Asian countries (Katsoyiannis et al. 2015b) and adsorption of arsenic onto iron oxides and iron oxide-coated sand in fixed-bed adsorption filters (Katsoyiannis et al. 2015a; Callegari et al. 2018) are being made to remove it so that people have safe drinking water supplies.

The use of fixed-bed adsorption media filters has gained considerable attention, especially for the treatment of waters with relatively low initial arsenic concentrations (i.e., in the range of 20 to 50 μg/L) due to the simplicity of operation and the efficiency of arsenic removal. These filters usually use the granular size of the adsorbents, i.e., higher than 250 μm (Thirunavukkarasu et al. 2003). However, micro-sized fractions (particle sizes of 1 to 250 μm) of granular ferric hydroxide that are very effective for adsorption of trivalent arsenic (As (III)) and pentavalent arsenic (As(V)) as reported by Usman et al. (2018) cannot be used in fixed-bed filters because of high clogging potential in fixed-bed adsorption filters, rapidly causing an increased pressure head, and thereby increasing energy costs and maintenance (Kalaruban et al. 2018; Vieira et al. 2017). However, considerable amounts of micro-sized fraction of granular ferric hydroxide (GFH), termed μGFH and tetravalent manganese feroxyhyte (TMF), termed μTMF are generated. μGFH is a by-product from industrial production of GFH, while μTMF produced during kilogram-scale production at the laboratory scale. These by-products might be applied in drinking water production not only to reduce the cost of water treatment, but also supply methods for by-product utilization. If fine fractions of adsorbent media will be added to a fluidized bed reactor, which treats water containing arsenic, adsorption of arsenic will take place. The treated water will subsequently be separated by low-pressure membrane submerged in a reactor. Recently, Hilbrandt et al. (2018) combined embedment of μGFH in a up-flow rapid filter with subsequent particle separator. The objective of particle separator is to retain the particles that might be washed out during the filtration process. However, they have tested the aforementioned process using cylindrical glass columns (2.4 × 100 cm) and its scaling up has not yet been performed, since rapid filter closely resemble the fixed-bed adsorption filter and fixed-bed filters packed with fine fractions of adsorbents are known to be prone to clogging during the filtration process in waterworks.

In the present study, the use of micro-sized fractions of the proposed adsorbents is investigated and its application is proposed in a combined unit with low-pressure membrane filtration to create an innovative hybrid treatment process, which could reduce the overall costs of treatment by using low-cost adsorbents and create a treatment process easily applicable from the household to the community based level. Low-pressure membrane processes, such as microfiltration (MF) or even ultrafiltration (UF), have a reasonable energy consumption and in general produce excellent hygienic quality treated water with a rather controllable membrane fouling at moderate capital costs (Katsoyiannis et al. 2013). A study of Drouiche et al. (2001) on economic performance of ultrafiltration membrane process indicated that a drinking water system (480 m^3^/day) treating surface water in the Kabylia region of Algeria incurred a total cost of 0.235$/m^3^ including capital, energy (0.010$/m^3^), membrane cleaning, and replacement costs.

Recent studies show that the removal of organic and inorganic pollutants from drinking water by a submerged membrane adsorption hybrid system (SMAHS) using micro-sized adsorbents is a promising technology (Vigneswaran et al. 2003; Kalaruban et al. 2018; Hilbrandt et al. 2019). The performance of a SMAHS depends on the adsorption capacity of the applied adsorbent media to remove specific pollutants, mode of adsorbent dosage (initially or continuously dosed to adsorption reactor), adsorption reactor configuration, and operating conditions including water matrix, hydrodynamic conditions such as air bubbling rate, water flux, feed water pH, temperature, etc. (Vigneswaran et al. 2003; Campos et al. 2000; Jia et al. 2009). High membrane water fluxes reduce costs due to large amounts of water being treated by small footprint installations. However, if due to increasing membrane water fluxes the hydraulic retention time in the adsorption reactor is rather short, the pollutant removal efficiency may decrease. Also, high fluxes may increase the rate of fouling on the membrane (critical flux phenomena). Nevertheless, applying aeration to the adsorbent suspension keeps the adsorbent particles completely dispersed in the reactor and helps to reduce the solid deposition on the membrane surface by the air scouring effect (Kalaruban et al. 2018; Stylianou et al. 2018; Choi et al. 2009). For example, in powdered activated carbon (PAC) adsorption-membrane filtration systems, PAC might be initially or continuously dosed into the adsorption reactor. For the “PAC initially dosed” mode, the required PAC is added into the reactor at the start of each filtration cycle. For the “PAC continuously dosed” mode, the PAC is continuously dosed into the adsorption reactor during the filtration cycle. Mathematical modeling using different adsorption kinetic models has shown that higher removal efficiency can be acquired with the “adsorbent initially dosed mode” due to higher adsorbent use efficiency with this approach (Campos et al. 2000; Chang et al. 2003).

The SMAHS and fixed-bed adsorption filters are dynamic and continuous flow treatment systems, which are more relevant to real-water treatment process than the static batch system of water treatment. The advantage of a SMAHS over conventional-bed filters is that micro-sized adsorbents exhibiting high surface area can be used in this system (Kalaruban et al. 2018). Moreover, the micro-sized iron oxyhydroxides are cheaper than the granular fractions. Currently, the costs (on dry basis) for GFH and μGFH materials was estimated to 9 €/kg vs. 1.6 €/kg, respectively (Usman et al. 2018). Another synergistic advantage of a SMAHS over fixed-bed absorbers is that low-pressure membranes achieve simultaneous removal of colloids, microorganisms, and suspended solids (Lebeau et al. 1998).

The primary objective of the study is to exploit the performance of micro-sized porous iron oxyhydroxides and also to identify the low-cost materials suitable for application in SMAHS for As(V) removal from a model drinking water source at varying operating conditions. The adsorbent efficiency was assessed in terms of volume of treated water until 10 μg As(V)/L (EU drinking water directives, US environmental protection agency (EPA) and World Health Organization (WHO) guideline value) was reached as well as the amount of arsenic adsorbed per unit mass of adsorbent. Moreover, the applied adsorbent media was characterized to better understand adsorption behavior. To the best knowledge of the authors, the performance of an adsorption-submerged membrane hybrid system using micro-sized fractions of conventional adsorbents to remove arsenic from drinking water under continuous flow operation is presented for first time in this study.

## Material and methods

μGFH was obtained from GEH Wasserchemie GmbH & Co, Osnabrück, Germany, and TMF was kindly supplied by colleague Manassis Mitrakas from Aristotle University of Thessaloniki (Tresintsi et al. 2013a). μGFH is a by-product generated during the industrial production of GFH, which is produced from a ferric chloride solution by neutralization and precipitation with sodium hydroxide. The ferric hydroxide precipitate was centrifuged and granulated by a high-pressure process (Thirunavukkarasu et al. 2003), while preparation of TMF involves the coprecipitation of FeSO_4_ and KMnO_4_ in a kilogram-scale continuous process. The kilogram-scale production in a laboratory two-stage continuous flow reactor includes the coprecipitation into the water of the iron source (FeSO_4_·H_2_O) at pH 4 and the manganese source (KMnO_4_), which is an oxidant for the process and also used to adjust the reaction’s redox to 850 mV (Tresintsi et al. 2013a). The generated fraction of μTMF during a laboratory-scale production of TMF was ca. 10%. Therefore, granular TMF (0.3–2 mm) was grounded to achieve abundant quantity of μTMF to apply in continuous flow experiments. All results were presented on a dry mass basis of both iron oxyhydroxides after drying at 105 °C for 24 h and subsequent cooling in a desiccator.

### Adsorbent characterization

Particle size distribution was determined by EyeTech™ analyzer (Combi, AmbiValue, the Netherlands). The iron content of the adsorptive media was determined by acid digestion. Briefly, 1 g of media (on dry basis) was added to 50 mL of 10% HNO_3_ in a glass beaker and the suspension was heated using a hot plate to boiling point. After 2 h, the iron oxide in the suspension was completely dissolved and the acid solution turned yellow (AWWARF 1993)). At this point, heating was ceased, the suspension after cooling was made up to 1 L with distilled deionized (DI) water, filtered through 0.45-μm filter, and the iron content determined by DIN 38406 method using a photometer (model UV-1700, Shimadzu, Germany). The surface area of the media was determined by nitrogen gas adsorption at liquid N_2_ temperature (77 K) using a surface area analyzer (Nova 4200, Quantachrome Instruments, USA) according to Brunauere Emmette Teller (BET) model. Six-point surface area measurements were employed to determine the surface area of the samples.

### Batch adsorption kinetic procedure

The kinetics of adsorption was conducted in model ground water (prepared according to National Sanitation Foundation (NSF) standards, termed NSF water hereafter) at pH 8 ± 0.1 with an adsorbent dose of 100 mg/L and a As(V) concentration of 190 μg/L by shaking Schott flask (2 L) containing test solution (1 L) at 150 rpm using a platform shaker. NSF water has the following composition: 252 mg NaHCO_3_, 12.14 mg NaNO_3_, 0.178 mg NaH_2_PO_4_·H_2_O, 2.21 mg NaF, 70.6 mg NaSiO_3_·5H_2_O, 147 mg CaCl_2_·2H_2_O, and 128.3 mg MgSO_4_·7H_2_O in 1 L of DI water (Simeonidis et al. 2017). The standard solution of As(V) was H_3_AsO_4_ in HNO_3_ (0.05 mol/L) with a concentration of 1 g/L. It was obtained from Merck chemical GmbH (Darmstadt, Germany). The experiments for each adsorbent were performed in duplicate.

Samples were taken at specific time intervals for a period up to 360 min. Upon sampling, samples were filtered with 0.45-μm (PES membrane) syringe filters and the filtrates were stored and analyzed at pH 2 using hydrochloric acid for total arsenic. A Perkin-Elmer atomic absorption spectrometer with a Perkin-Elmer Graphite Furnace Tube atomizer was used to measure the arsenic concentrations (Bower 1992). Argon gas was used to atomize the samples. The instrument setup parameters were 380 mA lamp current, detection at a wavelength of 193.7 nm, 0.7 nm silt width, and peak area as measurement mode.

### Submerged membrane adsorption hybrid system

**Fig. 1:**
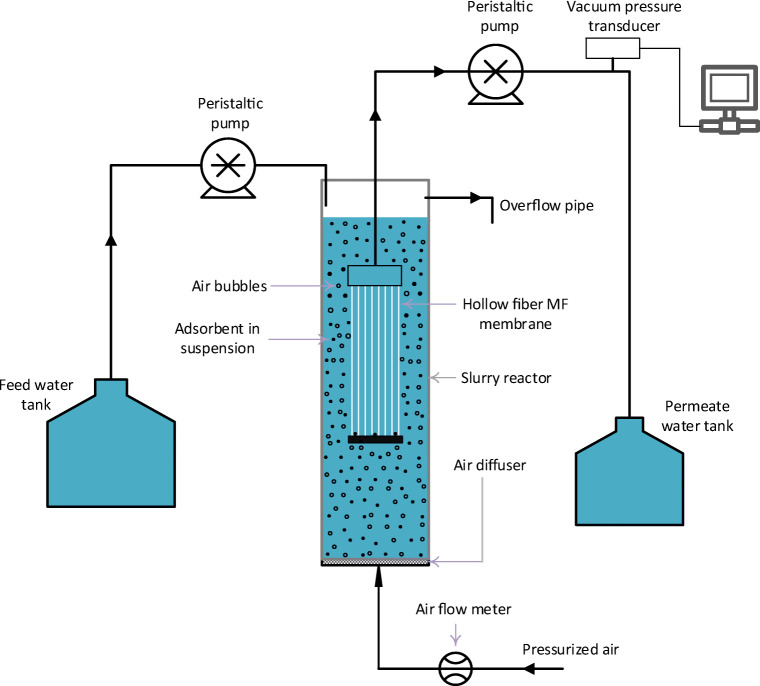
Schematic diagram of the submerged membrane adsorption hybrid system (SMAHS)

A self-assembled MF membrane module was made with hollow fiber outside-in PVDF-type membrane (Microza microfilter, Pall membrane) with specifications of 0.1 μm nominal pore size and 0.018 m^2^ was used in a SMAHS to separate the loaded adsorbent particles (Fig. 1). The inner and outer diameter of hollow MF fiber is 0.7 and 1.3 mm, respectively. The feed solution was prepared using NSF water spiked with As(V) to adjust a concentration of either 190 μg/L or 380 μg/L, and the pH of the solution was maintained at 8 ± 0.1.

The SMAHS experiments were carried out in a continuous flow operation. The adsorbents (1 g and 5 g each) were initially added to As(V) contaminated NSF water (1 L) in the slurry reactor and the MF membrane was submerged into the reactor. A peristaltic pump was used to feed the influent water to the slurry reactor and permeate was drawn from the reactor through a second peristaltic pump. The flow rates of both feed and permeate pumps were maintained identical and in order to keep the water level in the reactor constant throughout the experiments. Air was entered continuously from the bottom to keep the adsorbent particles in suspension and to generate scoring on membrane surface. In the absence of air, adsorbent particles could settle at the bottom of the slurry reactor and thus circumventing close contact between adsorbent and As(V) species. The transmembrane pressure was measured by a signal conditioned precision vacuum pressure transducer (423SC15D-PCB, Sensortechnics). The data were collected automatically by a data logger.

In most of studies (Adham et al. 1993; Hashlamon et al. 2017; Qu et al. 2018), the adsorption process is chosen as a pre-treatment. However, in this system, the iron oxyhydroxides were added directly to the slurry reactor. Consequently, As(V) adsorption and separation of arsenic-loaded adsorbent particles by MF membrane take place simultaneously in a single reactor.

## Results and discussion

### Characterization of adsorbents

Table 1 summarizes the physicochemical data derived by own analyses for both applied adsorbents.

**Table 1:** Main physicochemical characteristics of used adsorbent media

Media	Moisture content (%)	Fe content (wt%)	BET surface area (m^2^/g)	Pore volume (mL/g)	Mean particle size (μm)	Surface charge density (mmol OH¯/g)	Isoelectric point (IEP)
μGFH	~ 50 ± 2	59.8	283 ± 3	0.28	78.4	0.9^a^	7.8 ± 0.2^a^
μTMF	~ 5	44.5	178 ± 8	0.35	40.0	2.7^b^	7.2 ± 0.1^c^

^a^Usman et al. (2018), ^b^Tresintsi et al. (2014b), ^c^Tresintsi et al. (2013a)

As it can be seen in Table 1, both materials present a quite high Fe content and surface area. The results are similar to results of other studies. The determined BET surface area and Fe content of TMF were 187 m^2^/g and 38.1 wt%, respectively (Tresintsi et al. 2013a), while the BET surface area of μGFH reported by Hilbrandt et al. (2018) is 304 ± 5 m^2^/g. Both adsorbents have quite high Fe content, which is important regarding their adsorptive capacity, since the adsorption of arsenic takes place mainly because of the iron-based adsorption sites. In particular the adsorption of As(V) is believed to be dominated by (monodentate and bidentate) inner-sphere complexation between media surface groups and adsorbing moelcules. These types of surface complexes are restricted to ions such as arsenic that have a high affinity for surface sites and can bind to the media surface through covalent bonding (Essington 2015). The possible ligand exchange reaction for adsorption of As(V) by iron oxide-based adsorbent may include (Banerjee et al. 2008):

1 $$ {\mathrm{H}}_2{{{\mathrm{AsO}}_4}^{-}}_{\left(\mathrm{aq}\right)}+\mathrm{Fe}-{\mathrm{OOH}}_{\left(\mathrm{s}\right)}\to \mathrm{Fe}-{\mathrm{H}}_2{\mathrm{AsO}}_{4\left(\mathrm{s}\right)}+\mathrm{OH}\bar{\mkern6mu} $$

Regarding the surface area of the adsorbents, μGFH has considerably higher BET surface area than μTMF. This might play an important role on the adsorption efficiency of arsenic, acting synergistically to the very high Fe content. Both adsorbents have an isoelectric point at values above 7, in particular at 7.2 for μTMF and 7.8 for μGFH. The IEP value of the solid adsorbents plays a critical role as at solution pH values lower than the IEP, the overall surface charge of the adsorbent is positive. Given the fact that As(V) species are anionic over a broad pH range (i.e., from 3 up to 9) (H_2_AsO_4_^−^ and HAsO_4_^2−^), their adsorption onto porous (oxy) hydroxides (μGFH and μTMF) takes place mainly via electrostatic (Coulombic interaction) forces as well as ligand exchange reactions (Lewis acid-base interactions) to form monodentate and bidentate inner-sphere complexes (Banerjee et al. 2008). In the present study, the feed solution pH was much higher than the isoelectric point (IEP), which indicates that Coulombic interaction was not expected to be the main mechanism for As(V) adsorption and it is adsorbed onto iron oxyhydroxides by the formation of monodentate and bidentate inner-sphere complexes. Generally, it is presumably believed that porous nature of iron (oxy) hydroxides leads to As(V) adsorption at internal iron complexation sites (Sinha et al. 2002; Badruzzaman et al. 2004).

### Effect of contact time

**Fig. 2:**
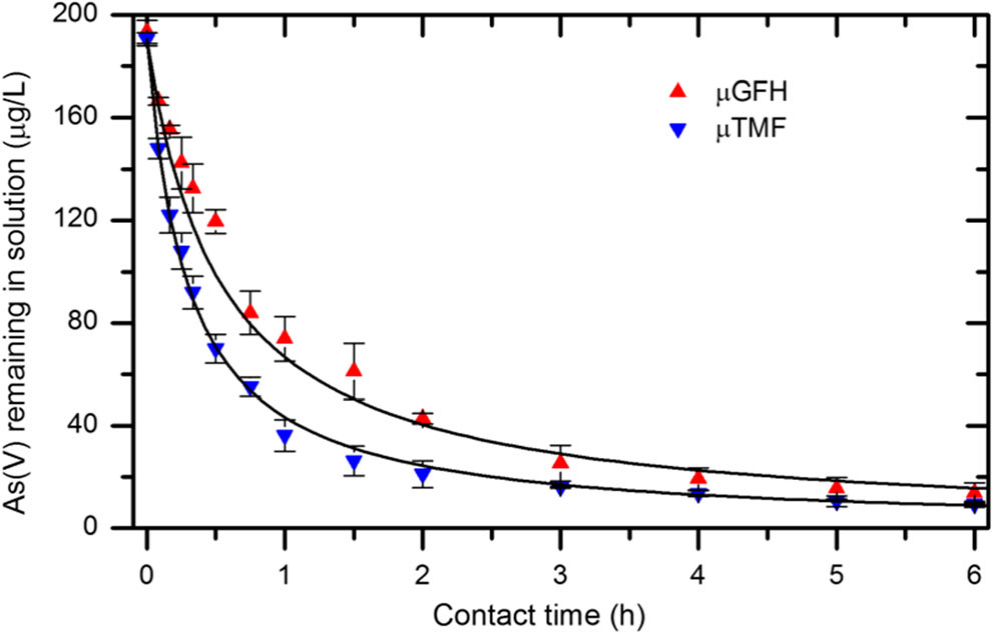
Effect of contact time on As(V) adsorption rate onto adsorbents in NSF water (*n* = 2). Solid lines represent the fitting using second-order adsorption kinetic model. Experimental conditions: Adsorbent dosage = 100 mg/L, initial As(V) = 190 μg/L, pH = 8 ± 0.1 and T = 20 °C, residual As(V) concentration for μTMF and μGFH is 9.5 and 13.8 μg/L, respectively

Batch adsorption kinetics experiments were conducted to study the effect of contact time on the rate of adsorption of As(V) onto adsorbent media (Fig. 2). The adsorption kinetic plots exhibit that initial rate of As(V) adsorption onto both adsorbents is rapid and removal rate increases with increasing contact time. At the end of the experiment (contact time of 6 h), about 95% of the As(V) was adsorbed onto μTMF relative to μGFH (93%). Due to the large concentration gradient between the bulk solution and media surface, the rapid initial removal rate of As(V) has been observed. This can be seen in Fig. 2 that about 70–80% of As(V) was adsorbed within the first 1 h of contact and only 20–30% additional As(V) adsorption has occurred in the following 5 h. Banerjee et al. (2008) reported that similar pattern during adsorption of As(V) onto GFH (with particle sizes between 0.32 and 2 mm) in ultrapure deionized water, but at a much higher initial arsenic to media ratio of 0.4 μg As(V)/mg GFH. In the present study, initial arsenic to media ratio is 1.9 μg As(V)/mg. It can be concluded that small size of μGFH favors faster removal rate of As(V) compared to GFH.

First- and second-order adsorption kinetic models were considered to analyze the removal rates of As(V) from aqueous solution. The simple forms of the first- and second-order kinetic models can be expressed as follows:

The first-order kinetic model:

2 $$ \ln \left(\frac{{\mathrm{As}}_{\mathrm{t}}}{{\mathrm{As}}_{\mathrm{o}}}\right)=-{k}_1\mathrm{t}, $$

The second-order kinetic model:

3 $$ \frac{1}{\mathrm{A}{\mathrm{s}}_{\mathrm{t}}}-\frac{1}{\mathrm{A}{\mathrm{s}}_{\mathrm{o}}}={k}_2\mathrm{t}, $$

where As_0_ is the initial concentration of As(V), As_t_ is the liquid phase As(V) concentration remaining in the solution at time t, and *k*_1_ and *k*_2_ are rate constants of first- and second-order kinetic models, respectively. The second-order kinetic model shows better fit to the kinetic data of As(V) adsorption onto iron oxyhydroxides as indicated by the higher correlation coefficient (R^2^) values in Table 2.

**Table 2:** The first- and second-order rate constants (*k*_*1*_ *& k*_*2*_) for the two adsorbents with different contact times

Media	First-order kinetic equation for				Second-order kinetic equation for			
	Contact time (≤ 3 h)		Contact time (≤ 6 h)		Contact time (≤ 3 h)		Contact time (≤ 6 h)	
	*k*_*1*_ (L/mg*h)	R^2^	*k*_*1*_ (L/mg*h)	R^2^	*k*_*2*_ (L/mg*h)	R^2^	*k*_*2*_ (L/mg*h)	R^2^
μGFH	1.06	0.68	0.53	0.86	0.010	0.95	0.014	0.99
μTMF	0.74	0.95	0.65	0.50	0.020	0.99	0.018	0.99

The calculated *k*_*2*_ values for the initial contact time of ≤ 3 h for μTMF and μGFH are 0.02 and 0.01 L/(mg*h), respectively (Table 2). The higher value of *k*_*2*_ for μTMF indicates the faster adsorption rate. This shows that As(V) removal occurred more rapidly with the μTMF which has a smaller particle size (Table 1). Though higher As(V) adsorption rate onto μTMF after a contact time of 3 h, μTMF removes 22% more arsenic within this time compared to μGFH.

### As(V) removal using submerged membrane adsorption hybrid system

The influence of various operating conditions on slurry reactor combining adsorption onto adsorbent media and a submerged MF membrane has been studied. In this unit, the added adsorbent media is used to remove pollutants, e.g., As(V) which is present in the source water, and at a second step, the submerged membrane functions as a complete barrier to arsenic loaded media particles. In the following section, the influence of several operational parameters have been studied, in order to define the optimum conditions for efficient operation of the hybrid treatment system.

### Hydrodynamic conditions/influence of air bubbling rate

The influence of bubbling on the adsorption process has been studied at bubbling rates 1.25, 2, and 3 L_air_/(min. L_slurry_). Air was transported from an air cylinder by PVC tubing to a sintered glass diffuser to generate fine air bubbles.

The adsorption process normally follows four consecutive steps (Jia et al. 2009): (1) external diffusion from bulk solution to liquid film, (2) diffusion in the liquid film surrounding the particle surface, (3) surface diffusion along the adsorbent inner surface, (4) adsorption of pollutant onto the active sites in the micropores. Among these four steps, the air bubbling rate will have an effect on the first two steps. Jia et al. (2009) reported during adsorption of Atrazine on PAC that mass transfer in the liquid film surrounding the adsorbent particle is very sensitive to air bubbling rate, and therefore, optimum air bubbling rate should be achieved for better removal of pollutant via adsorption in SMAHS.

**Fig. 3:**
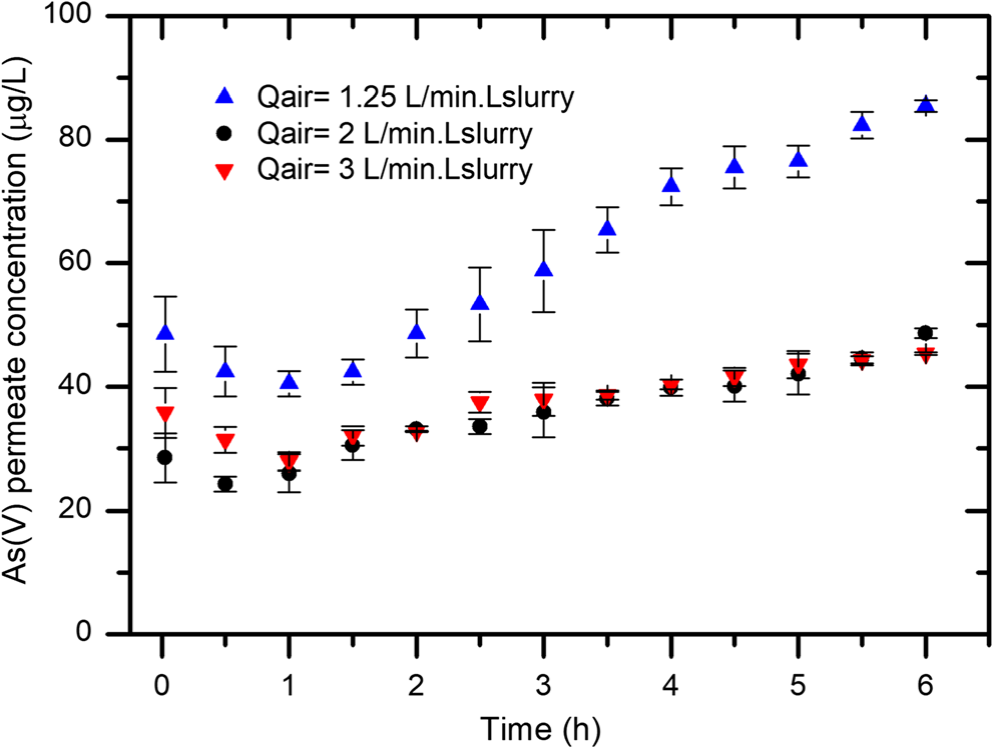
As(V) concentration in the permeate water over time in a  SMAHS with μGFH for varying air bubbling rates with As(V) = 190 μg/L, adsorbent dosage = 1 g/L, membrane flux = 20 L/(m^2^.h), feed solution pH = 8.0 and permeate pH = 8.0–8.2

Figure 3 shows that at all three air bubbling rates, As(V) removal efficiency of about 80% was achieved after approximately 5 min. At air bubbling rates of 2 and 3 L/min, an increase in the As(V) concentration with time was slow and As(V) removal efficiency of over 70% was achieved in a 6 h long continuous flow experiment. However, in case of 1.25 L/min, the increase in As(V) concentration with time was comparatively faster than that of 2 and 3 L/min, and at the end of 6 h, the As(V) permeate concentration was approximately 70 μg/L (just over 60% removal efficiency) at air bubbling rate of 1.25 L/min, while at 2 L/min, the As(V) permeate concentration was around 1.5 times lower than that of at 1.25 L/min air bubbling rate. It is concluded that air bubbling rate affects the arsenic concentration profile over time and reveals positive effect on adsorption process with increasing air bubbling rate from 1.25 to 2 L/min. Further increase in bubbling to 3 L/min did not noticeably enhance As(V) adsorption rate. According to Jia et al. (2005), there is a limit to bubble-induced mass transfer. This indicates that hydrodynamic conditions at ≥ 2 L_air_/min for As(V) mass transfer are optimized and a further increase in air bubbling rate will not promote the adsorption rate. It can also be concluded that air bubbles produced by means of sintered glass diffuser keep the adsorbent in suspension, therefore promotes the contact between adsorbent and adsorbate.

### Influence of adsorbent dosage

**Fig. 4:**
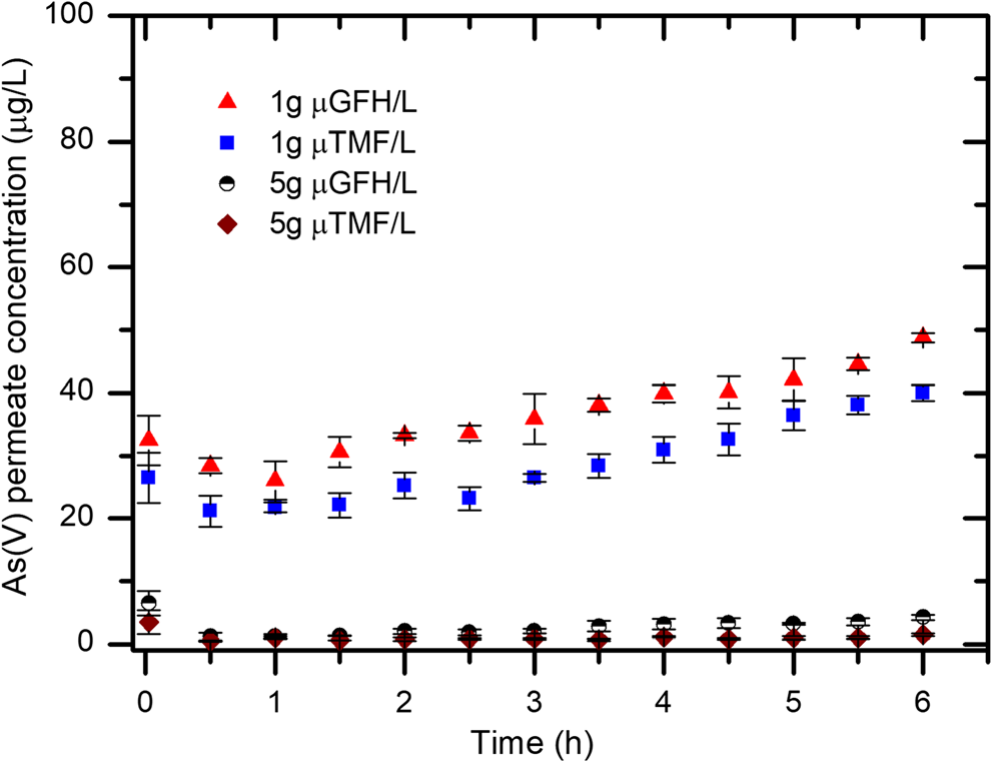
As(V) concentration in permeate over time in a SMAHS with μGFH and μTMF for adsorbent dosages of 1 and 5 g/L with initial As(V) concentration of 190 μg/L, air bubbling rate = 2 L/min and permeate pH = 8.0–8.3

Once the effect of the air inflow rate was quantified, the next important parameter in continuous operation units is the adsorbent dosage. In this section, the evaluation of the adsorption media in SMAHS was studied in terms of its ability to decrease the As(V) permeate concentration below the drinking water guideline value of 10 μg/L (termed Q_10,SMAHS_ hereafter), rather than to enhance the maximum capacity (Q_max_) and/ or higher removal efficiencies, which provides marginal information on ability of a specific adsorbent to reach guideline value set by EU drinking water directives, US environmental protection agency (EPA), and the World Health Organization (WHO). At an adsorbent dosage of 1 g/L, over 80% As(V) removal efficiency was obtained but As(V) concentration in the permeate exceeded the desire 10 μg/L WHO guideline value. Therefore, the amount of adsorbent initially dosed into the slurry reactor was increased to 5 g/L from 1 g/L to guarantee the As(V) permeate concentration below the WHO guideline value of 10 μg/L (Fig. 4).

It is pertinent to mention that by increasing the adsorbent dosage a slight increase to the pH value of the permeate (ranging between 8.0 and 8.3) was observed which can be attributed to the release of hydroxyl ion during adsorption of As(V) onto hydrous iron oxyhydroxide-based adsorbent (Eq. 1).

While at the dosage of 1 g/L of adsorbent initially dosed to the reactor, a slight difference in the As(V) adsorption efficiency between the two adsorbents was observed, at the dosage of 5 g/L, both adsorbent removed almost completely arsenic, and final concentrations were very low even up to the end of the experiment, i.e., after 6 h. Furthermore, at the dosage of 1 g/L, there is a continuous increase in the As(V) permeate concentration with time which starts to be evident even from the first hour of the experiment, most likely because of the gradual exhaustion of the adsorbent sites. In the case of 5 g/L, only after 4 h of the experiment, a slight increase in the permeate concentrations starts to be detected, but in all measurements, the As(V) permeate concentration was below the 10 μg/L.

However, it was found in this work that at an adsorbent dosage of 1 g/L, both adsorbents failed to meet the guideline value of 10 μg/L for arsenic in drinking water, as indicated at the EU Directive 98/83/EC. In the first case, the dosed adsorbent corresponds to 0.19 mg As(V)/g of adsorbent while in the second case, the ratio is 5 times lower, thus equals to 3.8 × 10^−2^ mg As(V)/g Adsorbent, under optimized conditions of air bubbling. During removal of nitrate in a submerged membrane adsorption system using ion exchange resins at water flux of 15 LMH, Kalaruban et al. (2018) used a ratio of 8 mg NO_3_/g to lower the adsorbate concentration from 20 mg/L to less than 11.3 mg/L (roughly 7 mg/L) in the reactor or permeate concentration, which is the guideline value for nitrate. Under these conditions, both adsorbent failed to maintain the nitration permeate concentration below 11.3 mg/L after 3–4 h at a hydraulic retention time of 2.7 h in the reactor.

### Influence of hydraulic residence time

**Fig. 5:**
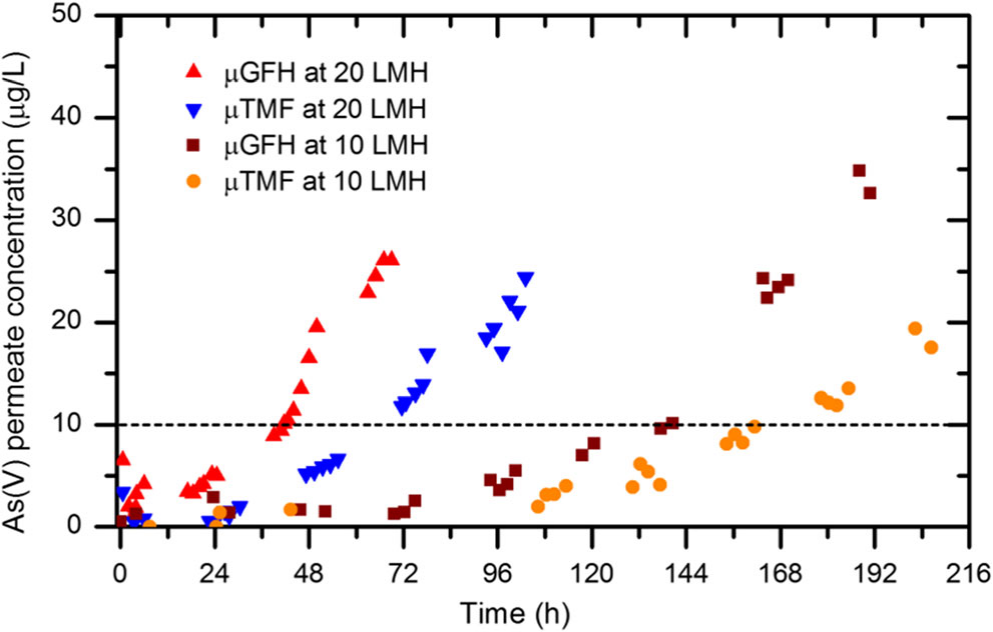
As(V) concentration in permeate vs. time for both media in a SMAHS at two different hydraulic residence times for adsorbent dosages of 5 g/L with initial As(V) concentration of 190 μg/L, air bubbling rate = 2 L/min and permeate pH = 8.0–8.3. The dashed line indicates the WHO guideline value for arsenic in drinking water

The residence time is a limiting factor in the slurry reactor as the adsorption kinetic plot (Fig. 2) shows that As(V) removal rate increased with increasing contact time. Therefore, the influence of hydraulic residence time on the performance of SMAHS has been studied at water fluxes of 10 L/(m^2^ h) and 20 L/(m^2^ h). Because the hydraulic retention times of As(V) in the slurry reactor were 2.8 h and 5.6 h at 20 L/(m^2^ h) and 10 L/(m^2^ h), respectively, accordingly, the initial 3 h and 6 h contact times of As(V) with media in adsorption kinetics were considered. Figure 5 shows the As(V) permeate concentration profiles derived by monitoring the SMAHS experiments using micro-sized iron oxyhydroxides. The results show that the addition of adsorbent results in a sudden decrease in As(V) concentration from 190 μg/L to a minimum value in the slurry reactor and after a day the As(V) concentration starts to increase with time. However, for the 10 LMH, As(V) concentration in the reactor stayed at minimum value for 3 days, and after which, the As(V) concentration in the permeate starts to rise but at a slower rate than 20 LMH.

It can be seen in Fig. 5 that As(V) permeate concentration profiles over time for both media in hybrid system are effected by the membrane water fluxes. However, the increase in As(V) permeate concentration over time for μGFH was rapid at both water fluxes. This shows that the μTMF is more effective than μGFH in adsorbing As(V) in the presence of competing ions.

The performance of media has been assessed in terms of the amount of As(V) adsorbed per unit mass of adsorbent and volume of treated water up to a guideline value of 10 μg/L. Q_10, SMAHS_ was calculated by dividing the area above the As(V) concentration curves by the initially added dry mass of adsorbent.

**Fig. 6:**
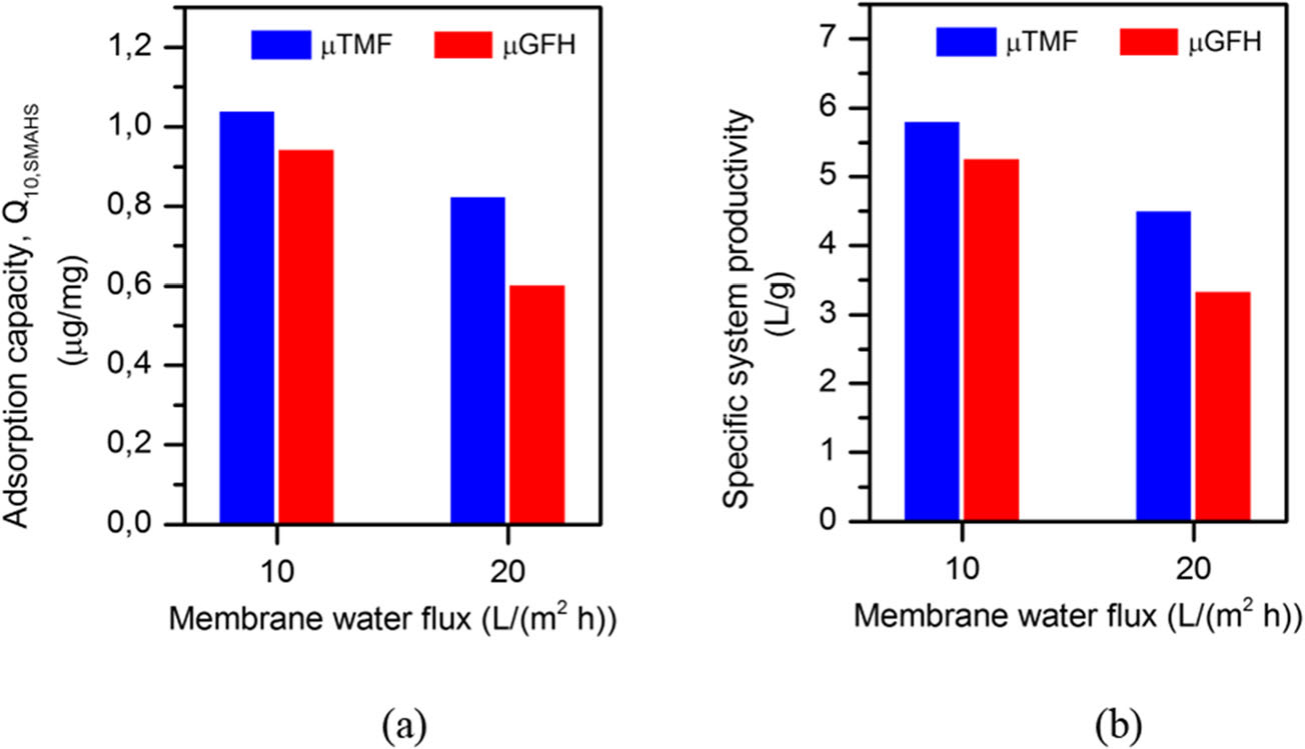
**a** Adsorption efficiency of both media in a SMAHS. **b** Specific system productivity at initial As(V) = 190 μg/L and permeate pH = 8.0–8.3

It can be seen from Fig. 6 that higher residence time (or lower flux of 10 LMH) increases the adsorption efficiency of media in removing As(V) from modeled groundwater, and thus is favorable, although it produces less treated water per unit time. The amount of As(V) adsorbed per unit mass of media has been decreased at 20 LMH. At 20 LMH, the recorded adsorption capacity of μGFH is 36.2% less than that of μTMF. The difference decreases to 9.9% at 10 LMH. This difference in Q_10,SMAHS_ at lower contact times can be explained by lower *k*_*2*_ values of μGFH (Table 2). These results showed that although the kinetics of As(V) adsorption was much faster for μTMF, the amount of arsenic adsorbed was finally similar for both adsorbent, and therefore, when the flux was decreased, the difference in arsenic capacity of both adsorbents—before As(V) permeate concentration reaches the 10 μg/L—has been reduced. This difference in Q_10,SMAHS_ value might also be due to greater surface charge density of μTMF (2.7 mmol OHˉ/g) than μGFH (0.89 mmol OHˉ/g). It is concluded that either particle size or surface charge characteristics or both have significant influence on practical adsorption capacity (Q_10,SMAHS_) for drinking water production observed in SMAHS, even though μGFH has higher BET surface area and IEP than μTMF.

The volume of water treated by unit mass of adsorbent to reach As(V) permeate concentration of 10 μg/L was calculated by defining as specific system productivity (SSP):

4 $$ \mathrm{SSP}=\frac{\mathrm{Q}\ {\mathrm{T}}_{10}}{\mathrm{V}\ {\mathrm{M}}_{\mathrm{ad}}} $$

**Fig. 7:**
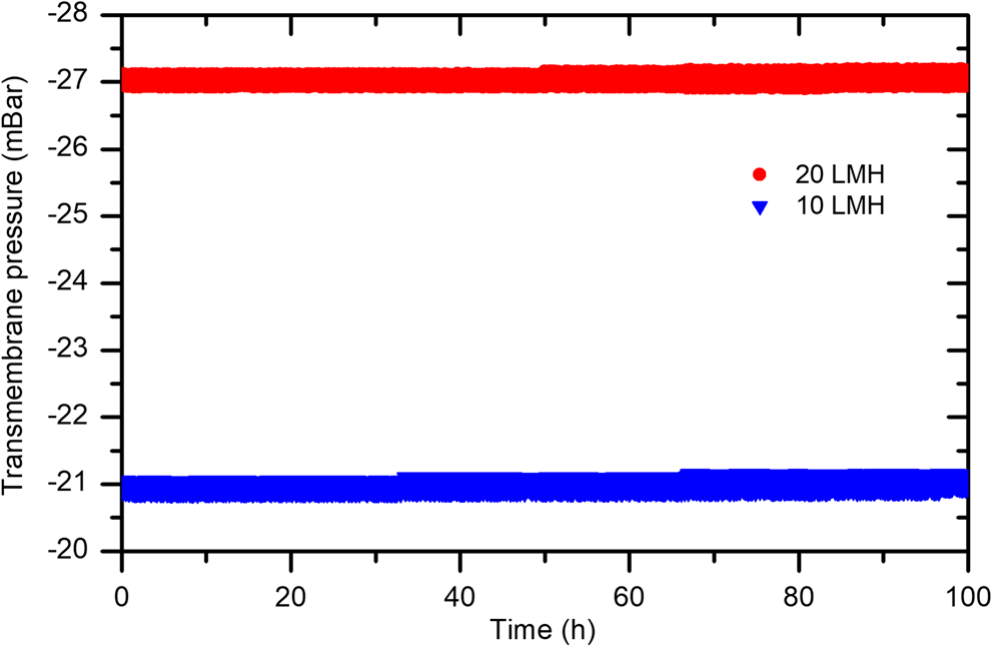
Transmembrane pressure profile during constant water flux in a submerged membrane adsorption hybrid system

Where Q is the volumetric flow rate at corresponding membrane water flux, T_10_ is time taken to reach 10 μg As(V)/L concentration in permeate, V is the liquid volume in the reactor, and M_ad_ is the mass of adsorbent initially dosed into the reactor. The results revealed (Fig. 6b) that the system productivity is higher at lower membrane water flux and vice versa. Furthermore, the recorded system productivity using μTMF is higher, compared with μGFH at both water fluxes. Figure 7 shows transmembrane pressure profile for constant flux filtration of 10 and 20 LMH. On the whole, it can be seen that the no fouling occurred during operation for both applied fluxes. It is recommended that membrane fouling behavior must be considered for long-term operation.

In summary, the As(V) adsorption capacity in SMAHS at contact time ≤ 6 h using μTMF is higher compared to μGFH at two different water fluxes. In a former study, we determined adsorption capacity (Q_10,batch_) through batch adsorption isotherm studies of 3.2 μg As(V)/mg and 3.3 μg As(V)/mg in NSF water for μGFH and at an equilibrium liquid phase concentration of 10 μg/L and at same pH with much longer contact time until adsorption equlibrium was reached (Usman et al. 2018). In batch adsorption tests, the recorded adsorption capacity of μTMF was 3.1% higher than μGFH. However, the obtained Q_10_ value for μTMF in SMAHS is 9.5% higher than the respective Q_10_ value for μGFH; this might be attributed to smaller particle size of μTMF, due to which adsorption rate is rapid as indicated by the adsorption kinetic plot of μTMF.

### Influence of initial As(V) concentration

The volume of water treated and amount of As(V) adsorbed per unit mass of adsorbent in the SMAHS using both adsorbents at varying As(V) feed concentrations and at identical flux is listed in Table 3.

**Table 3:** Volume of water treated and Q_10,SMAHS_ value for As(V) concentration < 10 μg/L for two adsorbent media at varying As(V) feed concentrations with adsorbent dosage = 5 g/L, water flux = 20 LMH, pH = 8 ± 0.1 and air bubbling rate = 2 L/min. L_slurry_

Media	Influent As(V) conc. (**μ**g/L)	Total operation time (h)	Total volume filtrated (L)	T_10_ (h)	Amount of As(V) adsorbed (mg)^a^	SSP (L/g)^a^	Q_10,SMAHS_ (μg/ mg)
μGFH	190	69	24.9	45.8	3.0	3.3	0.61
	380	68	27.5	43.0	5.8	3.1	1.15
μTMF	190	103	37.1	64.1	4.1	4.6	0.82
	380	102	36.6	57.0	7.7	4.1	1.54

^a^When As(V) concentration in permeate reached the WHO guideline value of 10 μg/L

As anticipated, specific system productivity using the micro-sized ion oxyhydroxides has been declined, when the As(V) feed concentration was increased from 190 to 380 μg/L. A reduction of 6% and 10% are recorded for μGFH and μTMF, respectively. However, Q_10,SMAHS_ value has been increased at the same water flux for both iron oxyhydroxides since the amount of arsenic entering the slurry reactor per unit time has been increased, and subsequently, the concentration gradient between the adsorbate in solution and the media solid surface has been increased and lead to higher Q_10_ value of both adsorbents. The time taken to reach the As(V) permeate concentration of 10 μg/L has been decreased in case of higher initial As(V) concentration of 380 μg/L. Hilbrandt et al. (2019) reported that during adsorption of phosphate onto μGFH in an adsorption-membrane hybrid system that a sharp slope of the breakthrough curve is favorable as it indicates less influence of film diffusion on adsorption. Due to which, a sharp increase in As(V) permeate concentration was observed and target contaminant concentration of 10 μg/L in the treated permeate with As(V) feed concentration of 380 μg/L was accomplished earlier than with 190 μg As(V)/L.

### Comparison of As(V) removal efficiency using SMAHS and fixed-bed filtration filter

The performance of the SMAHS using micro-sized iron hydroxides can be compared with laboratory- and full-scale fixed-bed adsorbers used for As(V) removal from the water via adsorption onto granular fractions of iron oxyhydroxide-based adsorbents as well as iron oxide-coated sand (Table 4). Tresintsi et al. (2013b) obtained higher As(V) adsorption capacity (i.e., Q_10_ value) of GFH, with particle size ranging between 250 and 500 μm, in rapid small-scale column test (RSSCT) than that of SMAHS using μGFH (1.7 vs. 0.95 μg/mg at 10 LMH or 0.61 μg/mg at 20 LMH). This difference in adsorption capacity might be explained because the two studies were conducted under different experimental setups and conditions, since the actual adsorption capacity of an adsorbent for a specific pollutant depends on experimental setups, water matrix, and solution pH.

**Table 4:** As(V) adsorption capacity of different iron oxyhydroxide-based adsorbents in a fixed-bed adsorption filter and SMAHS

Type of system	Test solution	pH	Phosphate conc. (mg/L)	Silica conc. (mg/L)	Influent As(V) conc.	Q_10_ (μg/mg)	Reference
GFH packed fixed-bed adsorber	Mitrousi (Greece) tap water	7.3	0.6	14	19	1.6	(Tresintsi et al. 2013b)
GFH packed RSSCT (lab-scale)	Thessaloniki (Greece) tap water	7.9	0.3	20	100	1.7	
GFH packed RSSCT (lab-scale)	Arizona (USA) groundwater	7.6	–	34	14	0.75	Westerhoff et al. (2005)
GFH packed fixed-bed adsorber	Arizona (USA) groundwater	7.8	–	39	34	1.6	
SMAHS using μGFH at 20 LMH	Artificial groundwater	8	0.12	20	190	0.61	This work
		8	0.12	20	380	1.15	
SMAHS using μTMF at 20 LMH	Artificial groundwater	8	0.12	20	190	0.82	
		8	0.12	20	380	1.54	
Iron oxide-coated sand packed fixed-bed adsorber	Groundwater	7.6	–	–	1000	0.002	Callegari et al. (2018)
Amyloid fibril – carbon hybrid membranes	Ultrapure water	7	–	–	239	0.27	Bolisetty et al. (2017)

Better performance of RSSCT packed with granular iron-oxyhydroxides for As(V) is associated to a larger concentration gradient between the adsorbate in solution and the media solid surface. The adsorbent in RSSCT is always in contact with the influent arsenic concentration, which results in a higher driving force over the whole adsorption process. However, in a SMAHS, the influent arsenic concentration is in contact with slurry, which has very low liquid phase arsenic concentration especially at the start of adsorption process when all adsorption sites are empty and arsenic removal occurred very rapidly, due to which the mass transfer driving force (concentration gradient) is very low compared to RSSCT. This is becoming more obvious, when the initial arsenic concentration increased to 380 μg/L. This provided more contact of arsenic species with the slurry of iron oxides and caused an increase in Q_10,SMAHS_ from 0.82 to 1.54 for μTMF and from 0.61 to 1.15 for μGFH respectively.

Regarding the experimental conditions, As(V) removal from the water via adsorption onto iron oxyhydroxides is known to be impacted by solution pH and presence of competing ions in the drinking water matrix. Westerhoff et al. (2005) investigated the arsenic adsorption in RSSCT packed with GFH using a different drinking water matrix with an As(V) influent concentration of 14 μg/L—where the concentration driving force might be in the same range as in SHAHS—even though the obtained Q_10_ value in RSSCT packed with GFH is lower than that of SMAHS using μGFH. This is possible because the both studies were conducted in a different drinking water matrix. A study by Amy et al. (2005) on effect of water matrix on arsenic adsorption reported a reduction of 70% in As(V) adsorption capacity onto GFH in the presence of 13.5 mg/L silica (SiO_2_) at pH 8 in batch adsorption tests. Similarly, 75% reduction in As(V) adsorption capacity was recorded in presence of 250 μg/L PO_4_^3−^ under similar experimental conditions.

Concerning the pH value, most commonly used adsorbents adsorb arsenic more effectively at pH values below IEP and their adsorptive capacities increase with decreasing pH (Tresintsi et al. 2012). During adsorption of As(V) onto iron oxyhydroxides, synthesized in laboratory at kilogram scale, in RSSCT, the Q_10_ value was increased from 2.8 to 6.8 to 10.7 μg/mg at 8, 7, and 6, respectively. This study demonstrated that by decreasing the solution pH by one unit from 8 to 7, the adsorption efficiency of adsorbent increased by 2.4 times. Similar results were obtained by Katsoyiannis and Zouboulis (2002), where As(V) adsorption was studied in fixed-bed columns using amorphous iron oxides as adsorbent; the bed volumes treated before arsenic concentration reached the 10 μg/L were increased as the pH decreased from 9 to 7. This is because as the pH decreases, the surface charge of the adsorbent becomes more positive and favors the adsorption of oxyanion species on their surface.

In summary, this difference in adsorption capacity between two studies could be attributed to water matrix as well as solution pH in addition to As(V) influent concentration, and therefore, it is relevant to mention that these factors play pivotal role while comparing the removal efficiencies of two water treatments systems for As(V) at roughly the same concentration driving force. From the above discussion, it is concluded that the removal efficiencies of both treatment systems are comparable for As(V) especially when the concentration driving force is higher in the slurry reactor and also taking into account the effect of the water matrix and solution pH. In one system, adsorbent media is fixed, while in hybrid system, adsorbent media is in suspension. Takin into account the experimental setup difference, it may be concluded that the use of this adsorption onto micro-sized iron oxyhydroxides followed by membrane separation might be an efficient solution for treatment of high arsenic content waters, as found in many countries including India, Bangladesh, Pakistan, Nepal, and China. This is because, at higher concentration driving force, the achieved Q_10_ values of the both applied adsorbents have been increased significantly.

Other types of fixed-bed filter that used low-cost sand coated with iron oxide to remove arsenic from groundwater, (Callegari et al. 2018), reported As(V) removal efficiency up to 99%. The iron oxide-coated sand fixed-bed filter could safely treat about 2–2.25 L water/L filter volume until 10 μg/L in the effluent was reached. With this volume of product water treated, the corresponding Q_10_ value of the iron oxide-coated sand is 0.002 μg/mg, which is at least three orders of magnitudes lower than the SMAHS using micro-sized iron oxyhydroxides in a complex water matrix. Additionally, the fixed-bed filter packed with iron oxide-coated sand needs around 2.5 h to reach stable arsenic effluent concentration (below 10 μg/L), from a 1000-μg As(V)/L initial concentration. However, in a SMAHS using low-cost micro-sized iron oxyhydroxides, the arsenic permeate concentration immediately reached arsenic concentrations below the drinking water guideline value starting from a 380-μg As(V)/L initial concentration. Moreover, the fined-sized adsorbent can be used in a SMAHS in addition to simultaneous removal of colloids, microorganisms, and suspended solids. Furthermore, the examined hybrid system is a relatively simple, effective option to treat arsenic-contaminated water and can find its application in decentralized water treatment system. Structural costs of the hybrid system are quite low, and the energy demand of pumps is relatively low that could be provided by solar photovoltaic panels.

The SMAHS performance can also be compared with similar studies using very advanced nanomaterials to achieve As(V) removal. The study of Bolisetty et al. (2017) shows that amyloid–carbon hybrid membranes containing 10% (by weight) amyloid fibrils indeed diminished the As(V) concentration in ultrapure water within the drinking water guideline value, but the adsorption capacity is only 0.27 μg/mg for As(V), and thus is almost 3 times lower than that of μGFH and μTMF in SMAHS, even without the presence of competing ions, showing that in the hybrid treatment system proposed in this study, the critical factor for optimized performance is the iron-based materials.

Considering environmental sustainability  of  process, spent adsorbent might be regenerated using an integrated procedure proposed by Tresintsi et al. (2014a). They have employed low-cost MgO to regenerate the used iron oxyhydroxides during As(V) removal in a fixed-bed adsorption filter. This approach combines the As(V) leaching (leaching step) and adsorption of leached As(V) onto MgO (adsorption step) under strong alkaline environment (0.05 N NaOH) in a continuous recirculation configuration. The MgO (below 3 wt%) is generally used in clinker which is a major constituent of cement. Arsenic-contained MgO can be successfully incorporated in commercially concrete products without any secondary pollution. Therefore, it can be concluded that the examined hybrid treatment system can be used without potential health risk in the process of waste handling.

## Conclusions

Two low-cost micro-sized iron-based oxyhydroxides were proved to be efficient in removing As(V) from artificial groundwater. The second-order adsorption kinetic described the adsorption kinetic data of both media well and confirms that As(V) adsorption kinetic was faster with μTMF than with μGFH under the same experimental conditions.

The SMAHS tests showed that both adsorbents can be applied by this approach without considerable fouling of MF membrane. Like batch adsorption tests, the adsorption kinetics of As(V) onto μTMF was faster than that of μGFH in hybrid system. Within the given SMAHS setup air bubbling rate of ≥ 2 L_air_/(min. L_slurry)_ ) was necessary to reach optimal conditions for the required mass transfer of As(V). Based on the continuous flow tests, the hybrid system benefits from a higher adsorbent dosage. The higher residence time of 5.6 h in the slurry reactor was favored for As(V) removal from contaminated water. As(V) adsorbent capacity in the hybrid system for As(V) increased and got almost double when the As(V) feed concentration increased to 380 μg/L from 190 μg/L at smaller hydraulic retention time. The Q_10_ value of hybrid system and fixed-bed adsorption filters were in similar ranges, taken into consideration the complete difference in the two compared units.

The media cost was estimated to be as low as 0.30 €/ L of treated water and media cost can be decreased significantly if pH of the raw water is lower than in the present study, i.e., between 6.5 and 7.5. Additionally, the media cost can be reduced remarkably by reusing spent iron oxyhydroxides, which can contribute to the low treatment cost of this hybrid system. In future research, the objective is the development of mathematical models to predict the As(V) permeate concentration profiles in a continuous flow hybrid system.
